# Evidence for Recombination as an Evolutionary Mechanism in Coronaviruses: Is SARS-CoV-2 an Exception?

**DOI:** 10.3389/fpubh.2022.859900

**Published:** 2022-03-17

**Authors:** Zisis Kozlakidis

**Affiliations:** International Agency for Research on Cancer, World Health Organization, Lyon, France

**Keywords:** COVID-19, recombination, evolution, viral recombination, coronavirus, SARS

## Introduction

The ability of RNA viruses to exhibit high rates of mutation and replication has been proven for over half a century and has been well documented with the advent of -omics technologies during the last two decades. These high rates of mutation relative to their hosts, allow them to evolve through the genomic evolutionary space, to broaden their variability and in some cases may afford them to acquire advantageous phenotypes in response to environmental pressures, e.g., anti-viral treatments, the latter changes can then become established in the particular evolutionary lineage of the virus (1, 2). Two additional, distinct but not mutually exclusive types of genetic exchange operate in RNA viruses, as a mechanism to acquire advantageous genomic changes, as well to be able to purge accumulated deleterious mutations. These are: firstly re-assortment, for viruses with segmented viral genomes such as Influenza, where antigenic shift in Influenza A is a well documented occurrence (3, 4). The second mechanism is recombination, which can occur both in segmented (5, 6) and non-segmented viruses, when such a mechanism exists; effectively when a “donor” sequence is introduced into a single contiguous genome to produce a new recombinant one. There is much excellent, recent literature summarizing the current knowledge and characterization of recombination for different RNA viruses at a population level (7–10).

Specifically in the Coronavirus family, recombination has been observed previously on a number of genomic studies. For example, recombination was reported in the MERS-CoV species (11, 12), while further phylogenetic analysis of the MERS-CoV full-genome sequences revealed recombination signatures that defined at least five major phylogenetically stable lineages, all of which contained human and camel MERS-CoV sequences (13). Similarly, for SARS-CoV there has been evidence for potential recombination events during its evolution (14, 15), as has also been suggested for human coronavirus HCoV-NL63, the latter exhibiting signs of having arisen from multiple recombination events from its nearest relative over its evolution (16, 17). As such, it is often reported that recombination is a normal consequence of coronavirus replication, required for the generation of the sub-genomic mRNAs and is also implicated in novel strain emergence (18–20).

## The Case of SARS-COV-2

In light of the above, it is of interest to consider the current evidence of recombination observed in the case of SARS-CoV-2. The SARS-CoV-2 virus was hypothesized to have emerged as a result of a recombination event between strains of beta-coronaviruses endemic to certain species of bats and pangolins (21), however this theory has invited intense debate as regards convincingly proving the proximal origin of the virus (22, 23). Specifically for the SARS-CoV-2 origins hypotheses, several authors provided arguments supporting the possibility that the SARS-CoV-2 genome is a chimera of the RaTG13 and Guangdong Pangolin coronavirus (i.e., a virus found in dead Malayan pangolins in the Guangdong province of China) (24–26) or in the place of the latter of close relatives of the bat CoV ZC45 and ZXC21 strains (24). Similarly, according to current hypotheses, evidence was presented that SARS-CoV-2 might be the result of recombination into RaTG13 from some unknown CoV strains (27). Such recombination events remain likely hypotheses at present, especially as in previous outbreaks intermediate hosts were implicated in the β-CoVs transmission (e.g., civets for SARS-CoV and camels for MERS-CoV) (20), suggesting that SARS-CoV-2 may have co-circulated with other coronaviruses in the wild in the same intermediary hosts, and also may have been transmitted to humans in this way.

To date, the SARS-CoV-2 genetic diversity increases slowly compared to other RNA viruses: given the many millions of infections globally and hundreds of thousands of genomes deposited in public databases (e.g., in the GISAID database) (28), there are only 7–8 major circulating clades observed, being identified based on multiple variants common to large numbers of isolates. It is this relative genomic stability of the circulating viral forms that allowed for the rapid development of effective vaccines and therapeutics, as well as supporting the deciphering of the SARS-CoV-2 pathology. However, inter- and intra-host recombination events in coronaviruses are well studied and evidenced to occur frequently (29, 30). As such the question arises on the lack of recombination events reported for circulating SARS-CoV-2 viruses. There have been a limited number of publications reporting any such recombination events (31–34).

It is becoming evident that while homologous recombination exists, recombinants seem to circulate at low levels for SARS-CoV-2 (31, 35, 36) with current estimates that at most 5% of circulating strains in the United Kingdom and USA are recombinants (36), or 16 recombinant sequences from the whole UK dataset of 279,000 sequences up to March 7, 2021 (31). On the other hand, it is also technically challenging to demonstrate homologous recombination when the genomic lineage evolution is driven by a limited number of single nucleotide polymorphisms. Furthermore, in order for homologous recombination to occur, the same cells within an individual need to be co-infected by genetically distinct viruses. Such co-infection of an individual requires that multiple viral lineages co-circulate within a population and, given the short duration of most SARS-CoV-2 infections, is most likely to be observed when virus prevalence is high in the population. Thus, the potential window of opportunity for the currently circulating SARS-CoV-2 variants is of limited time. To date, no heterologous recombination events have been reported, for example between SARS-CoV-2 and other co-circulating seasonal coronaviruses.

Having said that, the existing literature has demonstrated that the coronavirus proofreading exoribonuclease (nsp14-ExoN) is required to maintain the rates and loci of recombination generated during infection, and strongly supports that recombination mechanisms have been conserved across different evolutionary trajectories and host species specificity (18). Specifically, group 2a (MHV), 2b (SARS-CoV-2), and 2c (MERS-CoV) β-CoVs demonstrated many strong similarities in their patterns of recombination junctions across the genomes and in the types of recombined RNAs produced (18). Furthermore, during mixed infections of cell cultures with murine coronaviruses, at least 10% of progeny viruses were recombinants showing multiple independent recombination breakpoints (37). While such events appear unbiased in culture, in nature, events of recombination will be guided by natural selection pressures in regions with roles in host interactions. Among coronaviruses such areas of interest are centered in spike proteins (38). In light of the above, a particular case in SARS-CoV-2 can be hypothesized, with an upper ceiling of homologous recombination frequency (as evidenced by the experiments in culture) and potential recombination hotspots (spike protein) where the identification of such events would be most likely.

## Discussion

The coronavirus family is inclusive of many members, and the relative levels of recombination might be very different between different viruses even of the same family. In particular, the exact molecular mechanisms and determinants of RNA recombination in coronaviruses are only now becoming understood in greater detail, through the scaled-up surveillance and whole genome sequencing analyses (39), though the exact mechanisms and determinants of CoV recombination are not known (18). Additionally, for some outbreaks, there is little reason to suspect recombination, e.g., negative sense single-stranded RNA viruses are thought to recombine over evolutionary, not population-level, time scales (40). As more mutations and lineages of SARS-CoV-2 get fixed in the population and sequenced over a number of consecutive SARS-CoV-2 waves, a recombination event caused by a co-infection of a single patient with genetically distinct clades may lead to emergence of novel lineages, posing risks to the efficacy of future treatments.

Therefore, the following actions need to be considered: (i) a rapid and consistent surveillance of the sequenced SARS-CoV-2 genomes both for novel mutations and recombinations; (ii) a unified collection of genomic, epidemiological and clinical data; and (iii) further developed bioinformatics pipelines that allow for such recombination events to be detected within the limitations of the SARS-CoV-2 low genomic variation. While the first point is largely in place, the latter two points vary greatly between different geographic locations between and within countries. The UK presents a useful example in this respect, as the high rate of genomic surveillance and unified collection of genomic, epidemiological, and geographic data provide multiple lines of evidence for evaluating the identification of recombinant viruses. Establishing and operating such an integrated approach to viral surveillance on a consistent basis, remains critical to the ongoing identification of recombinants.

## Author Contributions

ZK conceived and wrote the Opinion manuscript.

## Author Disclaimer

Where authors are identified as personnel of the International Agency for Research on Cancer/WHO, the authors alone are responsible for the views expressed in this article and they do not necessarily represent the decisions, policy or views of the International Agency for Research on Cancer/WHO.

## Conflict of Interest

The author declares that the research was conducted in the absence of any commercial or financial relationships that could be construed as a potential conflict of interest.

## Publisher's Note

All claims expressed in this article are solely those of the authors and do not necessarily represent those of their affiliated organizations, or those of the publisher, the editors and the reviewers. Any product that may be evaluated in this article, or claim that may be made by its manufacturer, is not guaranteed or endorsed by the publisher.
